# Lipid oxidation-mediated non-enzymatic glycation and its impact on the digestibility of milk proteins

**DOI:** 10.3389/fnut.2026.1882160

**Published:** 2026-07-08

**Authors:** Haixia Yan, Chenyu Wang, Xiaofeng Lu, Ling Liu, Yixiao Shen

**Affiliations:** 1 College of Food Science, Shenyang Agricultural University, Shenyang, China; 2 College of Water Conservancy, Shenyang Agricultural University, Shenyang, China; 3 Liaoning Key Laboratory of Agricultural Product Processing Safety and Risk Assessment, Shenyang, China

**Keywords:** digestion characteristics, lipid oxidation, milk fat, non-enzymatic glycation, protein

## Abstract

**Background:**

This study explored the influence of milk fat on non-enzymatic glycation of proteins and protein digestibility by analyzing the accumulation of glycation products and changes in protein structure.

**Results:**

The contents of POV and MDA increased significantly on day 7 of storage in milk model groups with different milk fat concentrations (0, 2, 4, 6%) and the increase was more obvious in the high-fat (6%) group. In the high-fat group, the contents of glyoxal and methylglyoxal, the intermediate products of non-enzymatic glycation increased at a high level. The peak shift and the appearance of new peaks in the infrared spectrum further indicated enhanced glycation coupled with lipid oxidation. In addition, the decrease in fluorescence intensity, the increase in ultraviolet absorption, the reduction in surface hydrophobicity, and the change of protein secondary structure in the high fat group indicated obvious changes in the microstructure of proteins. The cleavage site of protease was reduced because of the alterations in protein advanced structure and the occupation of amino acids digestion sites, which subsequently reduced the digestibility of proteins. This phenomenon was more obvious in the early stage of gastrointestinal digestion.

**Conclusion:**

High levels of milk fat promoted non-enzymatic glycation by accelerating lipid oxidation, which in turn induced glycation, cross-linking, and aggregation of milk proteins. These structural changes further hindered the action of digestive enzymes, ultimately reducing the digestibility of milk proteins.

## 1 Introduction

Milk is a nutrient-rich substance, primarily composed of protein, fat, and lactose (1). In the dairy industry, milk serves as the principal source of production. According to the United Nations Food and Agriculture Organization, global milk production is projected to increase at an annual rate of 1.5% over the next decade. Within this growth, cow’s milk constitutes 81%, buffalo milk accounts for 15%, while goat’s milk, sheep’s milk, and camel’s milk collectively represent 4%. However, milk quality may deteriorate during storage and processing as a consequence of chemical reactions, including lipid oxidation, non-enzymatic glycation, and protein oxidation (2). Non-enzymatic glycation is typically initiated by the reaction between the carbonyl group of lactose and free amino groups, resulting in amino acid loss and decreased digestibility of milk proteins (3, 4). Furthermore, dicarbonyl compounds produced during lipid oxidation can further participate in glycation reactions, thereby accelerating the degradation process (5).

Milk contains approximately 29–34 g/L of high-quality digestible protein (6). However, its digestibility can be compromised by various processing and storage methods. Non-enzymatic glycation is a major factor that alters the structural integrity of milk proteins. Glycation intermediates, such as methylglyoxal (MGO) and glyoxal (GO), can modify the extent of protein hydrolysis in the gastrointestinal tract, thereby affecting both the bioavailability and nutritional functionality of milk proteins (7, 8). The reduction in protein digestibility is primarily attributed to the obstruction of enzymatic cleavage sites by glycation, which may occur through the direct blocking of lysine residues, the indirect masking of cleavage sites, and steric hindrance resulting from protein cross-linking (9, 10). In contrast, some investigations report that glycation-induced unfolding of whey proteins exposes additional enzymatic cleavage sites, thereby enhancing digestibility (11). The discrepancies observed across these studies may be attributed to variations in the types of proteins present in milk and their respective processing methods.

Fat oxidation also plays a crucial role in modulating the extent of milk protein glycation, thereby influencing protein digestion. However, the potential synergistic effects of milk fat oxidation on protein glycation and subsequent protein digestibility remain unclear. Therefore, this study was based on the hypothesis that protein glycation was promoted by lipid oxidation through the formation of dicarbonyl compounds, which in turn altered protein structure and ultimately affected protein digestibility. This research may provide a theoretical basis for optimizing nutrient preservation through the management of non-enzymatic glycation and fat oxidation reactions during milk processing and storage.

## 2 Materials and methods

### 2.1 Materials

Fresh milk was purchased from a local ranch (Liaoning, China) and transported on ice to the laboratory; GO (40%) and MGO (40%) were obtained from Sigma-Aldrich (St Louis, MO, USA); Pepsin (3,000 U/g) and pancreatin (4,000 U/g) were purchased from Beijing Solarbio Co., (Beijing, China); All other reagents used were analytical grade and were purchased from Shenyang Sinopharm Chemical Reagent Co., LTD. (Shenyang, Liaoning, China).

### 2.2 Sample preparation

The pasteurized milk sample was prepared referred to the methodology of Tan et al. (12). Briefly, raw milk was centrifuged at 4 °C and 4,000 rpm for 15 min to obtain skim milk and milk fat. The skim milk was evenly divided into four equal groups in 15 mL sterile covered centrifuge tubes, then, 0, 2, 4, and 6% milk fat was added in four groups, respectively. The mixtures were homogenized at 40 MPa according to the method of Zhang et al. (13) and then pasteurized at 65 °C for 30 min. Finally, four groups were stored at 4 °C and taken at 0, 1, 7, 14 and 21 d for analysis.

### 2.3 Determination of lipid oxidation products

#### 2.3.1 Peroxide value (POV)

The POV of milk samples was determined as described by Shantha & Decker (14) and Zhao et al. (15). Briefly, 50 μL of milk sample was added with 950 μL of chloroform:methanol (70:30) and centrifuged at 4 °C and 12,000 rpm for 10 min. Next, 100 μL of the lower layer solution was added with 900 μL of chloroform:methanol (70:30), 50 μL of potassium thiocyanate (300 g/L), and 50 μL of ferrous sulfate (3.5 g/L). The mixture was reacted in a microplate (96-well plates) at room temperature for 20 min, and the absorbance at 500 nm was measured using a microplate reader (BioTek Instruments Co., Ltd., American). The concentration of iron ions in the sample was calculated based on the standard curve in μg, and the POV was expressed as the mg equivalent of oxygen per Kg of fat.

#### 2.3.2 Malondialdehyde (MDA) content

The content of thiobarbituric acid (TBA) reactive substances in the milk was determined using the methodology of Zhao et al. (15). First, 0.5 mL of milk sample was mixed with 1 mL equal proportion mixture of trichloroacetic acid (150 g/L) and ethylenediaminetetraacetic acid (2 g/L) and centrifuged at 12,000 rpm and 4 °C for 10 min. Next, the supernatant was mixed with the same amount of TBA (0.288 g/L) and immediately subjected to a boiling water bath for 40 min. After cooling, the absorbance of the resulting sample was quickly measured at 532 nm. The MDA concentration in the sample was calculated based on the standard curve and expressed as the mass of MDA contained per Kg of fat.

#### 2.3.3 b* value of color

Briefly, 10 mL of milk from every group was weighed on a black plate, and the b* value of color was determined using a colorimeter (Konica Minolta, Osaka, Japan) at 0, 1, 7, 14, and 21 d.

### 2.4 Determination of non-enzymatic glycation level

#### 2.4.1 GO and MGO content

The contents of both MGO and GO were determined by ultra-high-performance liquid chromatography–tandem mass spectrometry (UPLC-MS/MS). A 20 g/L *o*-phenylenediamine solution was prepared in methanol. Next, 200 μL of milk sample was mixed with 400 μL methanol. Following centrifugation at 4 °C and 10,000 rpm for 30 min, 500 μL of supernatant was mixed with 500 μL of *o*-phenylenediamine solution, which was then derivatized at 4 °C for 12 h in the dark. The mixture was passed through a 0.22-μm organic filter membrane before UPLC-MS/MS detection. The mass spectrometry conditions were set referring to the method of Zhao et al. (15).

#### 2.4.2 Melanoidin content

The melanoidin content was determined following the method of Ding et al. (16) with slight modification. After the sample was diluted 200 times, the absorbance of the sample was determined at 420 nm using an ultraviolet–visible spectrophotometer (Beijing Puxin Co., Ltd., China). Ultrapure water was used as a blank control.

#### 2.4.3 Fluorescent advanced glycation end products (AGEs) content

The content of fluorescent AGEs in the sample was analyzed by fluorescence spectrophotometer (Hitach, Japan) after the sample was diluted 200 times. The excitation wavelength and emission wavelength were set to 347 nm and 440 nm, respectively. The slit width was 5 nm, and ultrapure water was used as blank control.

### 2.5 Determination of milk protein structure

#### 2.5.1 Circular dichroism (CD) spectroscopy

The circular dichroism spectrometer (Jasco Corp., Japan) was used to determine the protein secondary structure of milk samples. The milk sample was diluted with ultrapure water to a protein concentration of 0.1 g/L, and a spectral scan with a scanning range of 290–250 nm was performed at a scanning rate of 100 nm/min. The results of protein secondary structure were calculated by CDPro software.

#### 2.5.2 Fourier-transform infrared (FT-IR) spectroscopy

After freeze-drying in the vacuum, the sample was mixed with potassium bromide and pressed into thin slices. The spectral range of the FT-IR spectrometer (Nicoet460, Thermo Scientific, USA) was set to 4,000–500 cm^−1^, and 32 scans were performed at a resolution of 4 cm^−1^.

#### 2.5.3 UV–Vis absorption spectrum (UV–Vis)

The UV–Vis spectra of milk with different fat content were measured at 190–450 nm using an ultraviolet–visible spectrophotometer (Evolution 201, Thermo Scientific, USA) at a scanning rate of 2 nm/s. Phosphate buffer (0.01 mol/L, pH 7.0) was used as blank control.

#### 2.5.4 Surface hydrophobicity

The surface hydrophobicity was determined following the method described by Han et al. (17) with minor modifications. 1-Aniline-8-naphthalenesulfonate (ANS) was used as a probe to prepare 0.008 mol/L ANS solution with phosphate buffer (0.01 mol/L, pH 7.0). The milk samples were diluted to protein concentrations of 0.03125, 0.0625, 0.125, 0.25 and 0.5 g/L, and 50 μL ANS solution and 2 mL protein solution were mixed evenly. The fluorescence intensity was measured by a fluorescence spectrophotometer (Hitach, Japan) after standing in the dark for 5 min. The parameters were set as follows: slit width of 5 nm, excitation wavelength of 390 nm and emission wavelength of 470 nm.

#### 2.5.5 Three-dimensional fluorescence spectrum

Three-dimensional fluorescence spectra of milk samples were determined by fluorescence spectrophotometer (Hitach, Japan). The parameters were set as follows: slit width of 5 nm, emission wavelength of 200–800 nm, excitation wavelength of 200–500 nm, and scanning rate of 1,200 nm/min.

### 2.6 *In vitro* gastrointestinal digestion

In *vitro* simulated gastrointestinal digestion was performed according to the methods of Alison et al. (18) and Moreno-Montoro et al. (19) with slight modifications. The milk sample was centrifuged at 4 °C and 6,000 rpm for 15 min to obtain skim milk. Next, 10 mL of skim milk was preheated at 37 °C for 5 min and added with 10 mL of artificial gastric juice (2 g NaCl and 7 mL 2 mol/L HCl to 1 L); the pH was adjusted to 2.0 using 2 mol/L HCl. After digestion with 0.03 g of pepsin (4.5 U/mL) in a 37 °C thermostatic water bath for 30 min, 0.1 mL of artificial intestinal fluid (68.3 mL 84.7 g/L NaHCO_3_, 10 mL 22.2 g/L CaCl_2_·2H_2_O and 30 g bile salt were added to 500 mL) was added. The pH was adjusted to 7.0 using 1 mol/L NaOH. After adding 0.001 g of trypsin (0.2 U/mL) in a 37 °C constant temperature water bath for 2, 10, and 30 min to analyze the early-stage proteolysis, respectively, the reaction was immediately terminated by boiling the water in the water bath for 5 min. Hence, the gastrointestinal digestive juice of the sample was obtained, which was then subjected to sodium dodecyl sulfate-polyacrylamide gel electrophoresis (SDS-PAGE). These samples were named GD-0, GD-2, GD-10 and GD-30 according to the digestion time.

#### 2.6.1 Lysine and arginine content

The lysine and arginine content were determined as described by Neubeck et al. (20), with some modifications. The digested milk sample (2 mL) was centrifuged at 15,000 rpm for 15 min at 4 °C. The supernatant was mixed with 5% sulfosalicylic acid solution at a ratio of 1:1, and the mixture was centrifuged at 4 °C and 15,000 rpm for 20 min. Next, 800 μL of supernatant was passed through a 0.22-μm filter to determine amino acid content. The column temperature of the L-8900 automatic amino acid analyzer (HITACHI, Japan) was 57.0 °C, the reaction column temperature was 135 °C, the flow rates of pumps 1 and 2 were 0.40 and 0.35 mL/min, respectively, and the injection volume was 20 μL. The contents of amino acids in each milk group were obtained using the integral content of amino acids in the standard as a reference.

#### 2.6.2 Degree of protein hydrolysis (DH)

DH was determined as described by Nielsen et al. (21), with some modifications. The *o*-Phthalaldehyde (OPA) method was used to detect FAAs in the milk sample to analyze the DH of proteins. The milk samples of GD-0, GD-2, GD-10 and GD-30 were centrifuged at 4 °C and 10,000 rpm for 30 min, and the supernatant was isolated. Next, sodium tetraborate (19.05 mg) and SDS (500 mg) were dissolved in 375 mL of deionized water. 400 mg of OPA was dissolved in 10 mL of ethanol, and the OPA solution was quantitatively transferred to the mixed solution of sodium tetraborate and SDS. Finally, 440 mg of dithiothreitol was added, and the solution was diluted to 500 mL to prepare the OPA-mixed reagent. This OPA-mixed reagent (3 mL) and 400 μL of sample solution were mixed and allowed to stand for 2 min, then their optical density was measured at 340 nm using an ultraviolet–visible spectrophotometer (Beijing Puxin Co., Ltd., China). The DH in the milk sample was calculated referring to Nielsen et al. (21), as described in Equation 1.


$$\mathrm{DH}=h/h_{\mathrm{tot}}\times 100\%$$ (1)

*h* is the number of peptide bonds lysed per gram of protein after protein hydrolysis, in millimol per gram (mmol/g); *h_tot_* is the total amount of bonds, according to Nielsen et al. (21) selection of 8.3. L-lysine is the standard for DH determination.

### 2.7 Statistical analyses

All experiments were repeated three times, and three independent samples (n = 3) were set for each milk fat concentration. The results were expressed as mean ± standard deviation. One-way ANOVA test was used to analyze the differences between samples, and Pearson correlation coefficient was used to estimate the correlation between samples. *p* < 0.05 represented a significant difference. Origin 2022 software was used for drawing. Pearson correlation heat map was drawn at https://www.chiplot.online/.

## 3 Results

### 3.1 POV and MDA content in milk with different milk fat content

**Figure 1:**
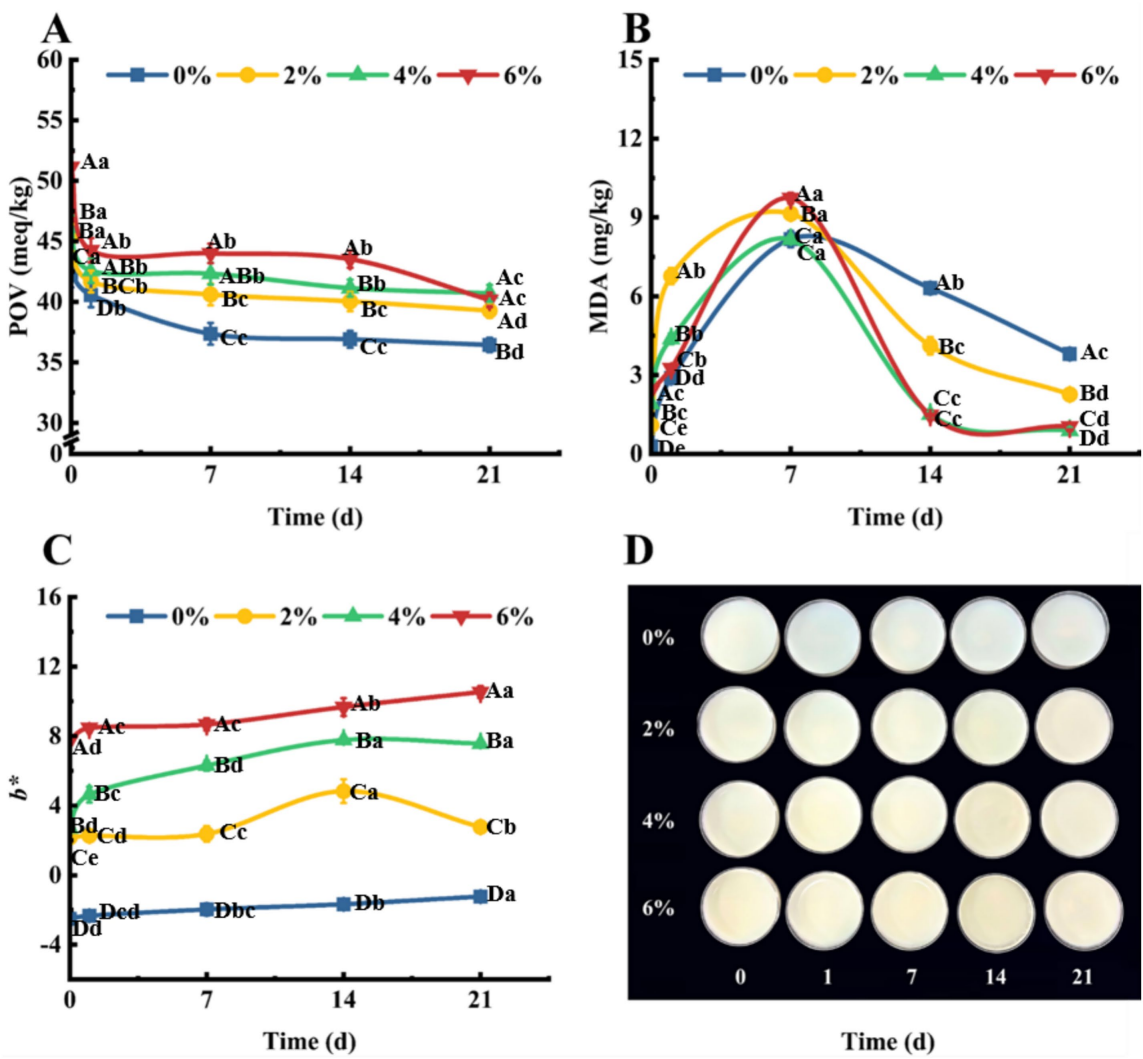
Contents of POV **(A)** and MDA **(B)**, b* **(C)** and image of storage milk **(D)** in 4 groups milk (0, 2, 4 and 6% milk fat) during storage at 4 °C for 21 days (*n* = 3). Uppercase letters represented the differences of four groups; lowercase letters represented the difference of the same group at different storage time.

Peroxide value (POV) and malondialdehyde (MDA) represent the primary and secondary oxidation products of the milk fat, respectively. During storage, POV decreased gradually in all groups (Figure 1A), with the 6% fat group exhibiting the most rapid decline. Concurrently, MDA levels initially increased and subsequently decreased as milk fat content rose. Notably, the 6% group showed higher fat oxidation at day 7 compared to the other groups (Figure 1B). Since MDA functioned as an intermediate in glycation reactions, its rapid fluctuations were closely associated with the formation of glycation products (22). These findings suggested that milk samples with higher fat content underwent accelerated lipid oxidation, forming a greater abundance of carbonyl compounds which served as critical intermediates for protein glycation.

### 3.2 Color change

The b* value, which quantifies yellow-blue chromaticity, is a key indicator of milk protein browning. Figure 1C illustrated the impact of differing milk fat contents on b* values. As storage time increased, the b* values in all groups except the 0% fat group exhibited an upward trend; notably, the 6% fat group demonstrated not only the highest b* value prior to storage but also maintained this peak during storage (Figure 1C). These results suggested that fat accelerated non-enzymatic glycation and the formation of brown products. Moreover, the initial color differences among the four milk groups were attributed to light scattering by milk fat globules (23). This finding was further supported by the actual milk colors shown in Figure 1D.

### 3.3 Glycation level in the milk with different milk fat content

**Figure 2:**
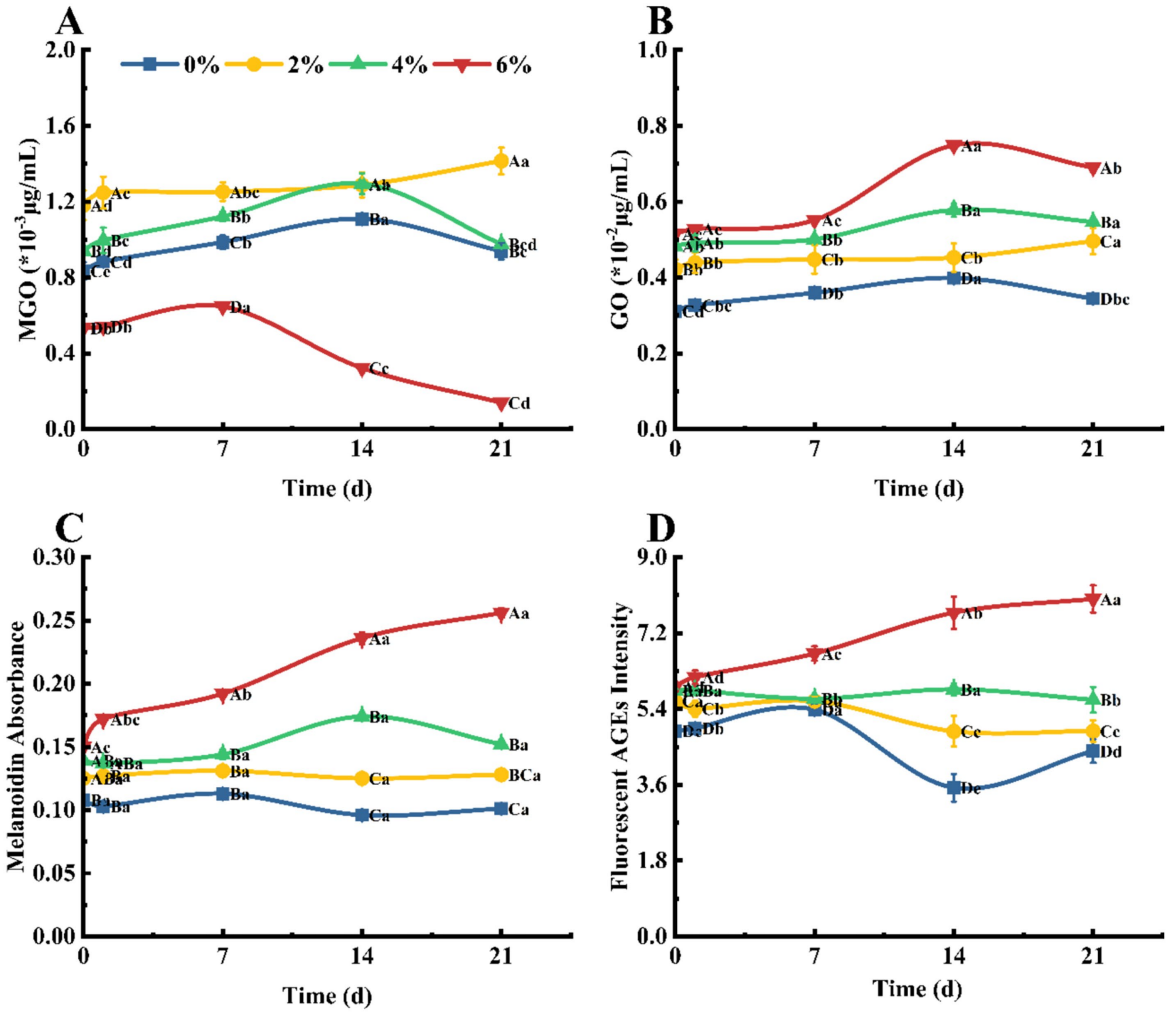
Contents of non-enzymatic glycation products MGO **(A)**; GO **(B)**, Melanoidin **(C)** and Fluorescent AGEs **(D)** in 4 groups milk (0, 2, 4 and 6% milk fat) during storage for 21 days (*n* = 3).

Some dicarbonyl compounds, such as MGO and GO, are unstable intermediates in Maillard reaction and lipid oxidation, tending to further form advanced glycation products (24). It can be seen from Figure 2A that the 0, 4, and 6% groups all exhibited rapid MGO accumulation. The 6% group reached its maximum near day 7, whereas the 0 and 4% groups peaked around day 14. The MGO content of 6% group was significantly lower than that of the other three groups (*P*<0.05), which might be due to its rapid conversion into advanced end products in the high-fat group. In addition, with increasing storage time, the GO content in the 0, 4, and 6% groups increased by 25.81, 18.75, and 35.29% at day 14, respectively, reaching their maximums (Figure 2B).

As milk fat content increased, MGO and GO contents showed a gradual upward trend, indicating that high milk fat content promoted the formation of dicarbonyl compounds and accelerated protein glycation in milk.

The dicarbonyl compounds are regarded as precursors of melanoidins in Maillard reaction (24, 25). In Figure 2C, there was no significant melanoidins generation in the 0 and 2% groups. However, the absorbance of melanoidins in the 4% group showed a peak on day 14, and the 6% group exhibited a significantly continuous increase (*P*<0.05). Apparently, 6% group did not peak during the 21-day storage period.

Fluorescence AGEs are used to evaluate non-enzymatic glycation end products with fluorescence characteristics. The changes of fluorescence AGEs content in milk with different milk fat contents during storage are shown in Figure 2D. In the 0% group, the fluorescence intensity fluctuated with storage time. The fluorescence intensity of the 2 and 4% groups changed slightly, whereas the 6% group increased significantly (*p* < 0.05), with the fluorescence intensity higher than that of the other three groups. This indicated that the increase of milk fat contents promoted the formation of fluorescent AGEs. Combined with Figures 2A,B, these results further suggested that the increase in milk fat content facilitated MGO and GO accumulation, which further promoted the accumulation of non-enzymatic glycation end products such as melanoidin and fluorescent AGEs.

### 3.4 Protein structure of milk with different fat content

#### 3.4.1 CD spectroscopy

**Figure 3:**
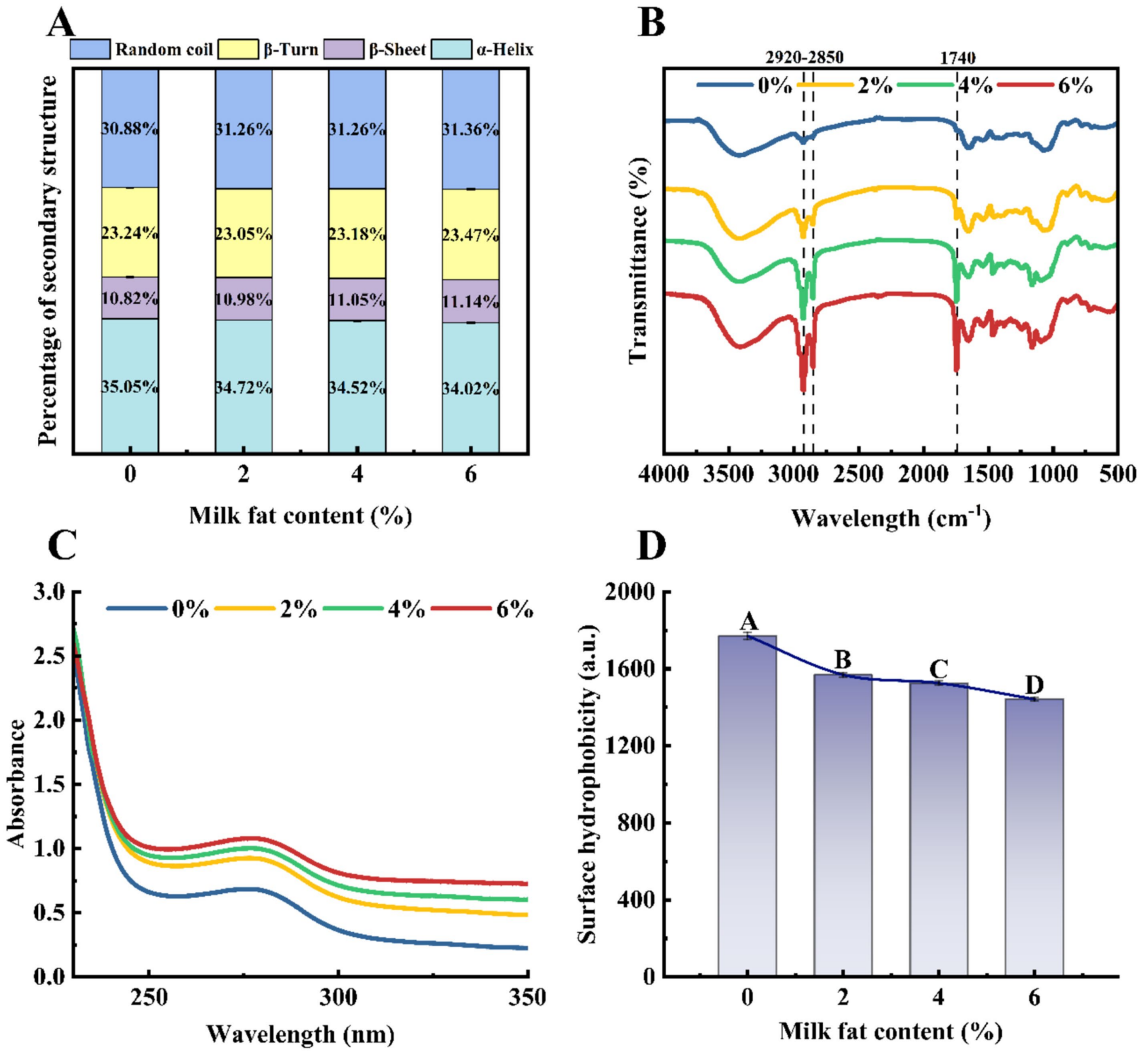
CD spectrum **(A)**; FT-IR spectrum **(B)**; UV–Vis absorption spectrum **(C)**; and Surface hydrophobicity **(D)** of 4 groups milk with different milk fat contents (0, 2, 4 and 6%) (*n* = 3).

CD spectrum of the protein mainly reflects its molecular conformational characteristics (26). The ratio of each secondary structure calculated by CDPro software were shown in Figure 3A. With the increase of milk fat content from 0 to 6%, the content of *α*-helix structure decreased from 35.05 to 34.02%, while the contents of *β*-sheet and random coil structure increased from 10.82 to 11.14% and 30.88 to 31.36%, respectively. The reduction in α-helix content means the relaxation of protein secondary structure, which is directly related to the increase of glycation level because protein glycation disrupted hydrogen bonds in the α-helix structure (27, 28). The breakdown of milk protein structure might intensify protein aggregation.

#### 3.4.2 FT-IR spectroscopy

The FT-IR spectra of milk samples with different milk fat contents are shown in Figure 3B. An increase in the milk fat content led to the redshift in the amide I, the 0, 2, 4, and 6% groups corresponded to 1656.77 cm^−1^, 1654.16 cm^−1^, 1648.94 cm^−1^, and 1646.33 cm^−1^, respectively. This shift could be attributed to the formation of Schiff base (C-N stretching) by glycation reactions (29). Meanwhile, the absorption peak intensity between 2,920 cm^−1^ and 2,850 cm^−1^ increased, representing the CH stretching vibration of -CH_3_ and -CH_2_ groups in the saturated structure when the milk fat was oxidized. Additionally, compared with the 0% group, a new absorption peak corresponded to the C=O bond appeared near 1740 cm^−1^ in the 2, 4, and 6% groups, with its intensity increasing as the fat content rose (30). This indicated that the increase of milk fat content promoted the formation of more carbonyl compounds.

#### 3.4.3 UV–Vis absorption spectrum

The characteristic absorption of tryptophan and tyrosine triggers a gentle absorption peak at about 280 nm, providing key information for protein structure research (31, 32). The changes in absorption peak position and height reflect alterations in the microenvironment of aromatic amino acid residues (33), indicating that milk fat content influences milk protein structure. As shown in Figure 3C, the absorbance at this peak gradually increased with higher milk fat content. Elevated absorbance indicated protein structure relaxation and changed in the microenvironment polarity surrounding internal amino acid residues, leading to greater exposure of hydrophobic chromophores (34, 35). This increased exposure of hydrophobic groups promoted hydrophobic aggregation between protein chains (36).

In addition, the absorption peak in the range of 260–320 nm was related to the characteristic region of melanoidins (37). It was showed in Figure 3C that a blue shift in the peak position from 277.02 nm in the 0% fat group to 276.04 nm in the 6% fat group, which suggested peptide bond cleavage and the exposure of hydrophobic groups (38). Similar observations have been reported for glycated bovine *β*-lactoglobulin (β-LG), which exhibited an absorption peak blue shift near 280 nm (39).

#### 3.4.4 Surface hydrophobicity

In order to further determine the exposure of hydrophobic amino acids, the protein hydrophobicity was evaluated by measuring the surface hydrophobicity (17). In Figure 3D, the surface hydrophobicity of the 0% group was the largest, which was 1770.63 a.u. With the increase of milk fat content, the surface hydrophobicity decreased significantly (*P*<0.05), dropping to 1442.40 a.u in the 6% group. The decrease in surface hydrophobicity of milk proteins was attributed to the binding of lactose to milk proteins. Specifically, the 6% fat group exhibited more pronounced protein structural changes, allowing more lactose to bind to the proteins, which led to an increase in hydrophilic hydroxyl groups. A similar phenomenon has also been observed in glycated rice bran protein and glutelin (40, 41). Glycated proteins are different from simple protein denaturation. Alterations in the protein microenvironment, such as the exposure of hydrophobic groups or an increase in surface hydrophilicity, modified the surface charge of the protein, leading to a diminished adsorption capacity for digestive enzymes (42).

#### 3.4.5 Three-dimensional fluorescence spectrum

**Figure 4:**
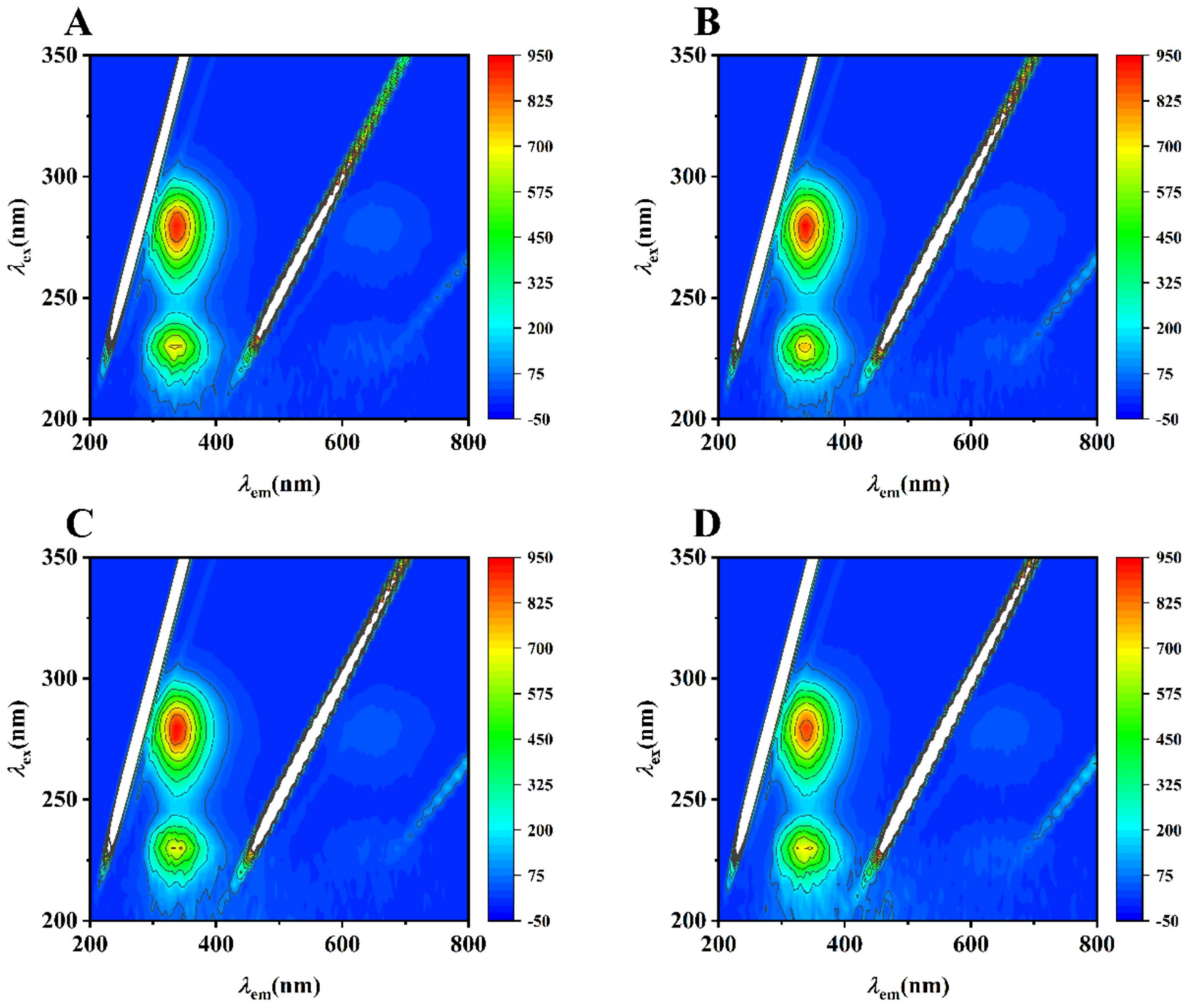
Three-dimensional fluorescence spectra of milk with different milk fat contents in 0% **(A)**, 2% **(B)**, 4% **(C)** and 6% **(D)** groups (*n* = 3).

In the three-dimensional fluorescence spectrum, Peak 1 primarily reflects the fluorescence characteristics of tryptophan (Trp) and tyrosine (Tyr) residues in proteins, while Peak 2 corresponds to the fluorescence signal of the polypeptide backbone. The occurrence of red or blue shifts in these peaks indicates interactions between proteins and their surrounding environment (43, 44). As depicted in Figure 4, distinct spectral intensities were observed among the four groups. According to Supplementary Table S1, with increasing milk fat content, the fluorescence intensities of both Peak 1 and Peak 2 initially increased and then decreased, likely due to alterations in the milk protein microenvironment and a reduction in protein hydrophobicity. Additionally, the observed red shift in emission wavelength suggested unfolding of the protein polypeptide chain, thereby enhancing interactions between milk proteins and lactose. This observation aligned with the decreased content of *α*-helix structures, as structural unfolding typically disrupted ordered secondary structures. Moreover, enhanced glycation levels indicated protein cross-linking and aggregation, processed known to negatively impact protein digestibility (45).

### 3.5 Changes in the protein digestion characteristics

#### 3.5.1 SDS-PAGE image

**Figure 5:**
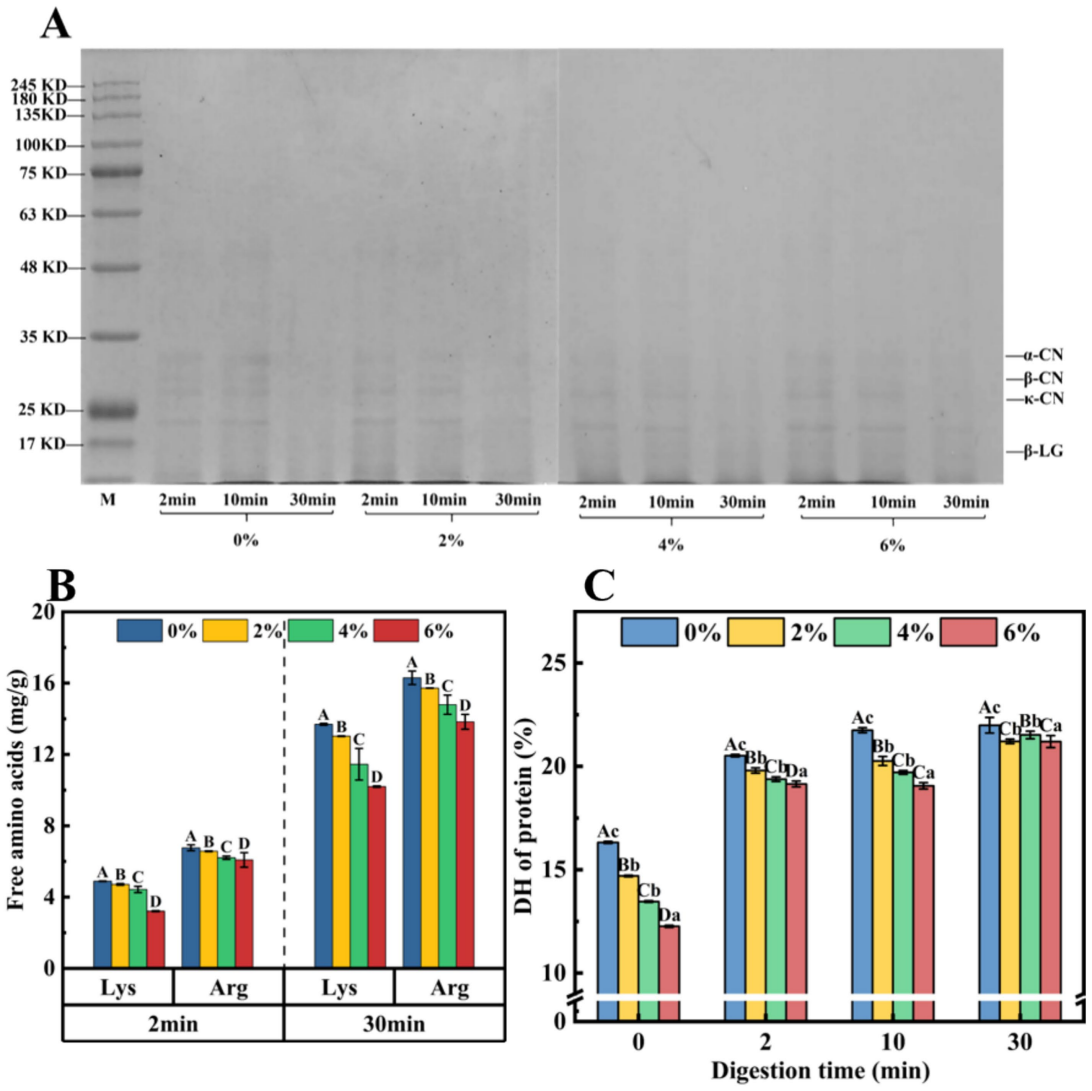
SDS-PAGE image **(A)**, lysine and arginine content **(B)**, degree of protein hydrolysis (DH) **(C)** of four groups of milk with different milk fat contents (0, 2, 4 and 6%) during simulated gastrointestinal digestion. The digestion time of the samples was 0, 2, 10 and 30 min (*n* = 3).

Figure 5A showed the SDS-PAGE image of milk proteins from groups with different fat contents during simulated gastrointestinal digestion. At 2 min of digestion, the high-molecular-weight (HMW) proteins in the 0 and 2% fat groups were rapidly hydrolyzed into low-molecular-weight (LMW) peptides. In contrast, distinct bands corresponding to HMW protein complexes and medium-molecular-weight (MMW) fragments formed during gastric digestion were observed in the 4 and 6% fat groups. With digestion times extended to 10 and 30 min, nearly all bands disappeared in the 0 and 2% groups, whereas some MMW bands remained in the 4 and 6% groups, with fewer LMW peptides generated. These results indicated that increasing milk fat content significantly impaired protein digestion, and that the digestibility of milk proteins was inversely related to both milk fat content and the level of protein glycation.

#### 3.5.2 Lysine and arginine content

As shown in Figure 5B, the contents of lysine and arginine in milk samples significantly decreased with increasing milk fat content. Among these, lysine served as a primary site for non-enzymatic glycation reactions (46). The lysine content in the 6% fat group was significantly lower than that in the other groups (*P* < 0.05), indicating a higher level of glycation in this group. Furthermore, lysine and arginine were the main cleavage sites for trypsin (47). When covalently modified through non-enzymatic glycation, these sites became specifically blocked, thereby reducing trypsin cleavage efficiency and ultimately decreasing protein digestibility (48).

#### 3.5.3 Degree of protein hydrolysis (DH)

DH reflected the extent to which proteins were degraded into peptides and amino acids. As shown in Figure 5C, DH increased with digestion time. At the same digestion time, samples with higher milk fat content exhibited a decreasing trend in DH, and the DH of the 0% fat group was significantly higher than that of the other groups (*P*<0.05). As previously described, the level of protein glycation increased with milk fat content. Consequently, the cross-linking and aggregation of proteins induced by glycation hindered enzymatic hydrolysis, as the steric hindrance of glycated proteins limited the accessibility of enzyme cleavage sites (11).

### 3.6 Correlation analysis

**Figure 6:**
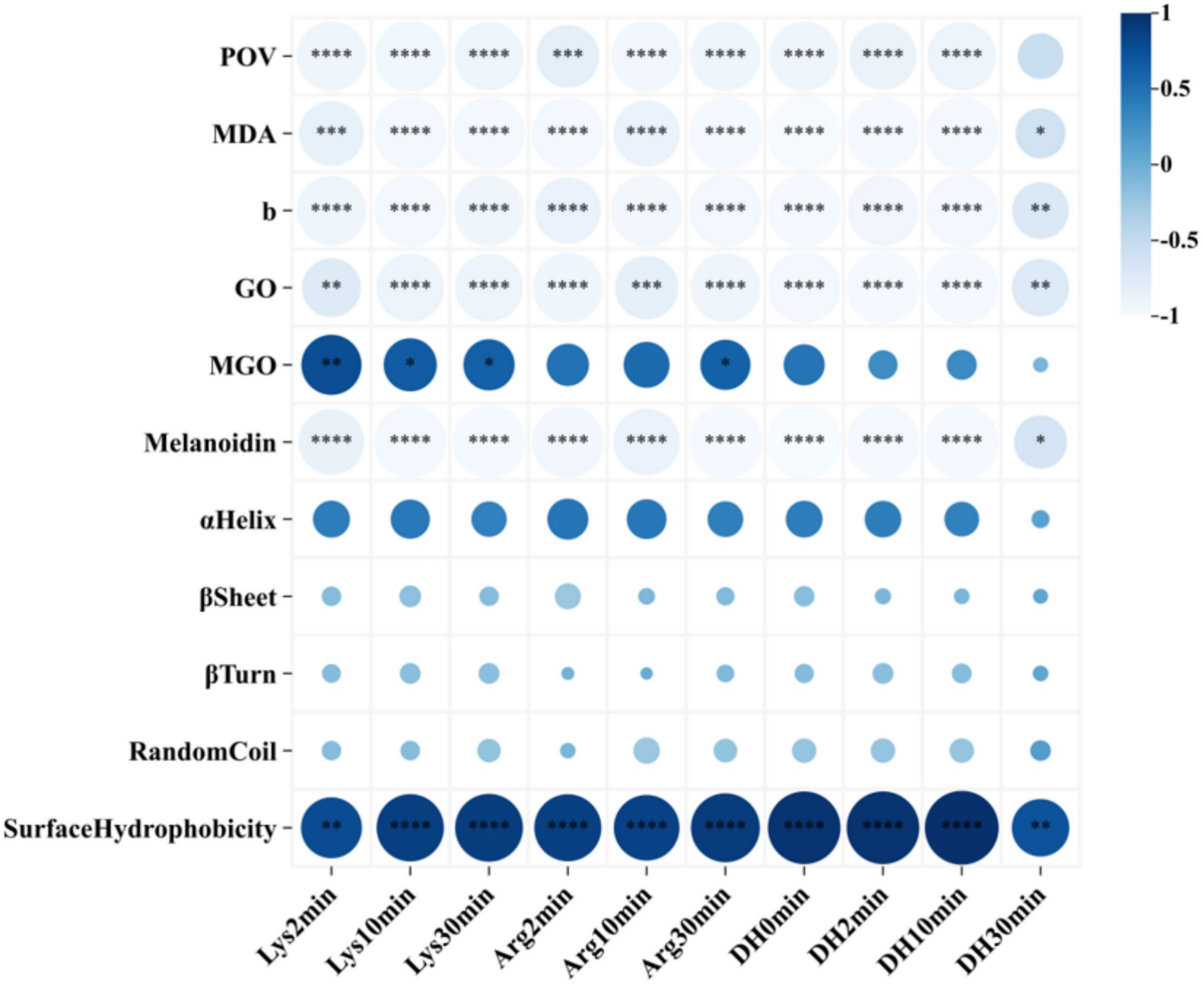
Pearson correlation analysis of lipid oxidation, non-enzymatic glycation, milk protein structure index and degree of protein hydrolysis of milk.

During gastrointestinal digestion, lysine content, arginine content, and DH which indicated the extent of digestion were significantly associated with non-enzymatic glycation indices, lipid oxidation markers, and changes in milk protein structure (Figure 6). They exhibited negative correlations with POV, MDA, b* value, GO, melanoidin content, *β*-sheet, β-turn, and random coil structures, and positive correlations with MGO, *α*-helix content, and surface hydrophobicity. These results suggested that the degree of milk fat oxidation was closely linked to glycation levels, which subsequently altered protein structure and impaired protein digestibility. Structural modifications, including changes in surface charge, the introduction of hydrophilic glycan chains, reduced accessibility of enzymatic cleavage sites, and extensive cross-linking, collectively contributed to decreased proteolytic efficiency. Notably, after 30 min of hydrolysis, oxidation and glycation indices no longer showed significant correlations with protein degradation, suggesting that as protein side chains were progressively cleaved and steric hindrance from glycated aggregates diminished, hydrolysis proceeded more readily. In other words, glycated proteins exerted a considerable impact primarily during the early stages of gastrointestinal digestion.

## 4 Discussion

Milk fat oxidation is a common deterioration during processing and storage of dairy products. This process induced non-enzymatic glycation and triggered structural changes in milk proteins. Therefore, it was hypothesized to affect the *in vivo* digestibility of milk proteins. To investigate the changes of milk digestion caused by milk fat during processing and storage, this study established the correlation between lipid oxidation markers (POV value and MDA) and protein glycation markers (b* value and melanoidin, etc.) with common intermediate dicarbonyl compounds shared by both lipid oxidation and glycation. The results indicated that the high-fat group exhibited higher levels of lipid oxidation, b* value, dicarbonyl compounds, and melanoidins. These findings confirmed that carbonyl compounds derived from lipid oxidation promoted glycation reactions. Studies have shown that dicarbonyl compounds cloud be formed by lipid oxidation and glycation reactions (49, 50). Our previous study has found that dicarbonyl compounds such as GO and MGO produced by lipid oxidation could serve as intermediates in the glycation reaction to promote the formation of AGEs such as carboxymethyl lysine in the glucose-lysine system (51). Furthermore, our previous research on the glycation of unsaturated fats demonstrated that fat oxidation generated active radicals and that electron transfer converted key amino acids (such as lysine) into free radical forms. These radicals subsequently participated in the formation of fructose lysine (FL) with reducing sugars via the Maillard reaction, thereby advancing the glycation pathway from FL to 3-deoxyglucose (3-DG) and ultimately to fluorescent AGEs (51). This result also suggested that in addition to the common intermediate products of lipid oxidation and protein glycation, free radicals were crucial intermediates linking lipid oxidation to the promotion of protein glycation.

An increase in milk fat content was associated with a deepening of lipid oxidation, which subsequently disrupted the secondary and tertiary structures of milk proteins. This disruption was shown as a decrease in *α*-helix content, an increase in *β*-sheet and random coil structure, elevated UV absorption peaks, and reduced surface hydrophobicity. Similarly, Chen et al. (52) observed a decrease in the α-helix content of myofibrillar proteins with the increase of MDA content, accompanied by increases in β-sheet and random coil structure. However, in contrast to this study, they reported a gradual increase in the surface hydrophobicity of myofibrillar proteins. This result was likely attributable to the covalent conjugation of fewer hydrophilic glycan chains to hydrophobic patches on the protein surface (52). Indeed, the degree of hydrophobicity depended on the extent of the glycation reactions. In the early stage of glycation, hydrophobic residues were exposed due to the disruption of the protein structure, resulting in an increase in surface hydrophobicity. Conversely, as glycation proceeded, the surface hydrophobicity decreased due to the introduction of more hydrophilic sugar chains (53). In this study, the degree of glycation increased significantly with the increase of milk fat content during pasteurization, and more hydrophilic sugar chains were introduced. Therefore, the surface hydrophobicity of the high-fat group was lower than that of the low-fat group.

Glycated milk proteins were prone to aggregation and cross-linking, which consequently affected their digestibility. In this study, HMW proteins mainly existed in the early stage of digestion, and gradually degraded into MMW and LMW proteins in the middle and late stages of digestion. The degree of protein hydrolysis was lower in the high-fat group compared to the low-fat group, which was consistent with Zhao et al. (8). They also found that glycation markedly reduced the *in vitro* digestibility of *β*-casein and β-lactoglobulin. In addition to glycation reaction, an increase in milk fat content was also considered to influence the structural stability and digestibility of protein through interfacial adsorption, spatial shielding effect, and lipid-protein interaction. For example, the adsorption of proteins to the oil–water interface could be promoted as the total surface area of fat globules increased, leading to significant changes in their advanced structures (54). Proteins could migrate from the aqueous phase to the lipid phase by hydrophobic interactions, forming protein-fat clusters, which in turn changed the spatial distribution of proteins (55). Moreover, the physical barrier formed by the interface layer hindered the contact between proteins and digestive enzymes. In addition, the alterations in the *ζ*-potential of the emulsion affected the adsorption efficiency of enzymes through electrostatic interaction, thus indirectly interfering with the enzymatic hydrolysis process of the protein (56). Although these phenomena were observed to exist between milk fat and proteins, since the samples were defatted prior to digestion in this study, the observed differences in digestibility among the various milk fat groups were mainly attributed to the glycation reaction.

In summary, non-enzymatic glycation, oxidative modification, and protein aggregation collectively induced structural changes in milk proteins. These changes ultimately reduced the digestibility of glycated proteins by masking protease cleavage sites and reducing substrate accessibility (9). As glycation progressed, the availability of lysine and arginine residues (the action sites of trypsin) was reduced by covalent modification, directly limiting enzymatic proteolysis. With further progression of glycation, the proteolytic efficiency was further reduced because the accessibility of digestive enzymes to the protein core was limited. This limitation arose from substantial steric hindrance induced by extensive protein cross-linking and the attachment of numerous hydrophilic glycan chains.

It should be noted that the aforementioned results were mainly based on protein structural changes and the effect of lipids has not been fully considered, which was a limitation shared by most current studies solely on the protein perspective. However, in the case of raw materials with high protein and high fat such as milk, an increase in milk fat content led to more intense oxidation, resulting in a rise in free radicals in milk protein and accelerating oxidative denaturation. Therefore, in addition to the decrease in protein digestion caused by the aforementioned structural changes, glycation and oxidation products (such as MDA) might alter the surface charge of milk proteins and weaken the electrostatic interactions between digestive enzymes and their substrates (52). Furthermore, based on the compositional and structural characteristics of milk proteins and lipids, the initial stage of glycation involved rapid interactions between whey proteins and lactose, occurring much faster than glycation involving casein (57). As milk fat oxidation progressed and glycation deepened, casein micelles gradually disintegrated and participated extensively in glycation, resulting in high cross-linking in dairy products (58, 59). Concurrently, the progressive destruction of the milk fat globule membrane by oxidation triggered milk fat aggregation and creaming. This process might promote protein aggregation and induce phase separation between milk proteins and fat, which exacerbated the reduction in protein digestion level. However, compared with the INFOGEST protocol, a lower final enzyme active and a shorter digestion duration were employed in this study. This adjustment was based on preliminary experiments, in which milk proteins from all groups were fully hydrolyzed after 2 h of digestion under high final enzyme activity. As a result, no differences in protein digestibility were observed among the groups. Since the primary objective of this study was to capture variations in protein digestion caused by different milk fat contents, rather than to simulate complete gastrointestinal transit, the final enzyme activity and digestion duration were reduced accordingly. This change made it possible to distinguish the differential effects of milk fat content on protein digestibility more clearly. Furthermore, to better observe the digestion levels across varying milk fat conditions, the composition of the digestive fluids was simplified following the methods described by Alison et al. (18) and Moreno-Montoro et al. (19). While this allowed valid internal comparisons, caution should be taken when extrapolating the findings to *in vivo* digestion.

## 5 Conclusion

In this study, the relationship between milk fat and protein glycation was systematically investigated, establishing a positive correlation between milk fat content and glycation level. The elevated milk fat content accelerated lipid oxidation, leading to the formation of dicarbonyl compounds and the accumulation of free radicals, which in turn increased the production of intermediate products of the glycation reaction. This accumulation further promoted the involvement of reducing sugars and induced denaturation, cross-linking, and aggregation of milk proteins. Moreover, the reduction in protein digestibility was collectively driven by changes in protein hydrophobicity and glycation sites, along with protein cross-linking and steric hindrance from sugar chains. Overall, milk represents a complex system in which oxidation and glycation processes are closely interrelated. Based on the above findings, future studies could be directed toward quantifying the specific dicarbonyl compounds generated during milk fat oxidation and clarifying their individual contributions to the glycation modification of major milk proteins such as *β*-lactoglobulin and caseins.

## Data Availability

The raw data supporting the conclusions of this article will be made available by the authors, without undue reservation.
