# Supplementary material for: Lipid oxidation-mediated non-enzymatic glycation and its impact on the digestibility of milk proteins
Source: Front Nutr. 2026 Jul 8;13:1882160. doi: 10.3389/fnut.2026.1882160 (PMC13436951; doi:10.3389/fnut.2026.1882160)
Supplement: Supplementary file 1 [file Table_1.docx]

Supplementary Material

# 1 Supplementary Table

## 1.1 Supplementary Table

**Supplementary Table S1** Three-dimensional fluorescence spectral parameters of milk protein with different milk fat contents

| Milk fat content（%） | Peak 1 | | Peak 2 | |
| --- | --- | --- | --- | --- |
|  | Peak  λex/λem  （nm/nm） | Intensity  F_0_ | Peak  λ_ex_/λ_em_  （nm/nm） | Intensity  F_0_ |
| 0 | 230/345 | 721.1±1.2^d^ | 280/335 | 916.7±2.2^c^ |
| 2 | 230/345 | 738.0±2.1^a^ | 280/335 | 949.1±2.5^a^ |
| 4 | 230/345 | 727.8±1.6^b^ | 280/340 | 933.0±4.3^b^ |
| 6 | 230/345 | 725.7±1.7^c^ | 280/340 | 880.0±8.01^d^ |

Note: The results with the different superscript letters in each row were significantly different (P<0.05).
